# Umbilical cord accidents

**DOI:** 10.1186/1471-2393-12-S1-A7

**Published:** 2012-08-28

**Authors:** Jason H Collins

**Affiliations:** 1 Pregnancy Institute, New Roads, LA, USA

## 

The Stillbirth Collaborative Research Network recently reported on the probable or possible cause of death of 512 stillbirths whose mothers consented to complete postmortem examination [1]. Umbilical cord accidents (UCA) represented 10% of stillbirths [1]. In Caucasians the UCA associated stillbirth rate was 13% and 4% in non-Hispanic black. 9% of stillbirths were due to hypertension and 8% due to other maternal medical disorders. A literature review places the UCA associated stillbirth rate at 15% [2]. These data bases do not include stillbirth due to several UCA pathologies such as: torsion, multiple cord entanglement and abnormal placental cord insertion. The main reason for these absences is the belief by some that these abnormalities do not cause actual death or recurrent stillbirth.

One of the first published accounts of an UCA in western medical writings was by William Smellie his Treatise on Midwifery in 1750, London, England: a nuchal cord associated stillbirth. One of the first published drawings of an UCA was by Andrew Bell in the Encyclopedia Britanica 1st edition 1769 Edinburgh, Scotland, depicting a fetal death with a combination of one nuchal cord, a body loop and a true knot (currently on the cover of the Royal College of Obstetricians and Gynaecologists (UK) brochure).

As UCA is a significant cause of death, JC argues it is now time for the focus to be on screening for UCA, managing UCA prenatally and delivery of the baby in distress defined by the American Congress of Obstetricians and Gynecologists as a heart rate of 90 beats per minute for 1 minute on a recorded non-stress test. The ability of ultrasound and magnetic resonance imaging (MRI) to visualize UCA is well documented. The 18-20 week ultrasound review should include the umbilical cord, its characteristics and description of its placental and fetal attachment. The American Association of Ultrasound Technologists has defined these parameters for umbilical cord abnormalities:

• Abnormal insertion

• Vasa previa

• Abnormal composition

• Cysts, hematomas and masses

• Umbilical cord thrombosis

• Coiling, collapse, knotting and prolapse

Umbilical cord evaluation with sonography includes the appearance, composition, location and size of the cord [3]. A normal cord has a single vein and 2 arteries that have a twisted, rope-like appearance. Absence of twisting often is associated with a decrease in fetal movement and a poor pregnancy prognosis.

Umbilical cord pathology is separate from placental pathology [4]. Developmentally the umbilical cord is fetal in origin not placental [5]. The umbilical cord originates from the “primitive ridge” of the embryo. There are paternal genetic elements influencing growth and development. To date there have been no reports of mosaicism in the human umbilical cord. The Human Genome Project has not reviewed cord genetics. There are eight different umbilical cord designs. None of these issues have been incorporated into a detailed prospective study of pregnancy and outcomes. Our current knowledge of the human umbilical cord and its influence on the fetus is limited. Interactions between the fetus and umbilical cord are becoming apparent due to studies of fetal behavior.

Hyperactivity is a fetal response associated with umbilical cord compression risk factors [6]. This fetal behavior may be related to intrauterine umbilical blood flow disturbance which stimulate the fetus to react reflexively and excessively. Animal studies (in rats and sheep) have reproduced forms of hyperactivity with cord compression. Hyperactivity may be a prenatal behavior capable of repositioning the fetus and relieving the compression. In the rat model, umbilical cord compression triggered lateral trunk curls, head tosses and foreleg extensions. In the sheep model intermittent umbilical cord compression triggered fetal hiccups. Hiccups occurring daily after 28 weeks, and greater than 4 times per day requires fetal evaluation. UCA should be looked for no matter how trivial it seems on ultrasound.

Fetal body movements have been studied with ultrasound over 24 hour periods [7]. These movements are unique between midnight and 6 a.m. Time of fetal behavioral observation (bedtime and midnight to 6 a.m.) may need to be included in any future stillbirth study. Fetal jerking movements and fetal hiccups may also be related to fetal blood flow disturbances especially cord compression. These maternal observations should be taken seriously and prompt an ultrasound review of the fetus looking for UCA.

Recent research into circadian rhythms may help explain why UCA stillbirth is an event between 2 a.m. and 4 a.m. Melatonin has been described as stimulating uterine contractions through the M2 receptor [8]. Melatonin secretion from the pineal gland begins around 10 p.m. and peaks to 60 pg at 3 a.m. Serum levels decline to below 10 pg by 6 a.m. Uterine stimulation intensifies and may be overwhelming to a compromised fetus, especially one experiencing intermittent umbilical cord compression due to UCA. Pregnancy Institute has documented over 1000 UCA stillbirths through patient interview that occurred during maternal sleep.

UCA are an important cause of stillbirth. It is now possible to identify UCA on ultrasound and test for the compromised fetus. As with gestational hypertension, screening for UCA is needed to possibly avoid thousands of stillbirths worldwide. If UCA is detected, the mother should be hospitalized and evaluated with ultrasound and fetal heart rate monitoring for at least 24 hours. If fetal behavior or the fetal heart rate is abnormal, the observation period should be extended and if necessary deliver the baby.
